# Supplementary material for: A conserved Pol II elongator SPT6L mediates Pol V transcription to regulate RNA-directed DNA methylation in Arabidopsis
Source: Nat Commun. 2024 May 25;15:4460. doi: 10.1038/s41467-024-48940-8 (PMC11127964; doi:10.1038/s41467-024-48940-8)
Supplement: Supplementary file 1 — Supplementary Information [file 41467_2024_48940_MOESM1_ESM.pdf]

A conserved Pol II elongator SPT6L mediates Pol V transcription to regulate RNA-directed DNA methylation in *Arabidopsis*

Yujuan Liu[1,2,3], Jie Shu[2,4], Zhi Zhang[1,2,3], Ning Ding[2,5], Jinyuan Liu[1,2,3], Jun Liu[4], Yuhai Cui[6,7], Changhu Wang[1,2,3*], Chen Chen[1,2,3*]

[1]State Key Laboratory of Plant Diversity and Specialty Crops, South China Botanical Garden, Chinese Academy of Sciences, Guangzhou, Guangdong 510650, China

[2]Key Laboratory of South China Agricultural Plant Molecular Analysis and Genetic Improvement & Guangdong Provincial Key Laboratory of Applied Botany, South China Botanical Garden, Chinese Academy of Sciences, Guangzhou, Guangdong, 510650, China

[3]University of the Chinese Academy of Sciences, Beijing, 100049, China

[4]Guangdong Academy of Agricultural Sciences, Guangzhou, Guangdong, 510640, China

[5]MOE Key Laboratory of Cell Activities and Stress Adaptations, School of Life Sciences, Lanzhou University, Lanzhou 730000, China

[6]Agriculture and Agri-Food Canada, London Research and Development Centre, London, Ontario, N5V 4T3, Canada

[7]Department of Biology, Western University, London, Ontario, N6A 5B7, Canada

[*]To whom correspondence should be addressed. Tel: +86 020-37252711; Email: wangchangh@scib.ac.cn (to Changhu Wang) and chenchen101@scbg.ac.cn (to Chen Chen)

Supplementary Fig. 1: SPT6L co-binds with NRPE1 at intergenic regions

a: Heatmaps of SPT6L, Pol II, and NRPE1 ChIP signals around peak center of all SPT6L peaks. The SPT6L peaks were clustered into two groups (genic and intergenic). The plotted regions are upstream and downstream 1 kb of peak center. The SPT6L and Pol II ChIP-seq data sourced from GSE108673. The NRPE1 ChIP-seq data sourced from GSE124546.

b: Pie charts showed the proportions of transposable elements (TE), gene, and others within SPT6L genic and intergenic peaks.

c: Heatmaps of NRPE1 and SPT6L ChIP signals around peak center of all NRPE1 peaks. According to the differential binding patterns of SPT6L at NRPE1 peaks, the NRPE1 peaks had been clustered into NRPE1-only and NRPE1-SPT6L overlapped peaks.

d: The plots of TE frequency within NRPE1-SPT6L overlapped and random peaks (defined in Supplementary Fig. 1c). Two types of randomizations were applied: randomly selected same amounts of peaks as that of NRPE1-SPT6L overlapped peaks in total NRPE1 binding sites (blue lines); randomly shuffled NRPE1-SPT6L overlapped peaks across entire genome (gray lines).

e: Pie charts indicated the proportions of different TE groups in *Arabidopsis* genome, NRPE1 binding peaks, and NRPE1-SPT6L overlapped peaks, respectively.

Supplementary Fig. 2: HMM model defined genome states and the interaction of Pol V and SPT6L

a: Confocal image examined the nuclear localization of NRPE1–GFP in 7-day after germination (DAG) roots; Scale bars, 20 μm.

b: Chop-PCR analysis of DNA methylation at *SN1*, *IGN23*, and *IGN25* in *WT*, *nrpe1*, and three transgenic lines of *nrpe1 ProNRPE1:NRPE1-GFP* performed by digestion with *Hae*III restriction endonuclease. Digested genomic DNA was amplified by PCR. Sequences lacking *Hae*III (*Actin2*) were used as loading controls.

c: Venn diagram showed the reproducibility of our identified NRPE1 binding peaks (in seedlings) with published NRPE1 peak list (inflorescence).

d: Heatmap showed six genomic states defined by ChromHMM according to differential enrichment of SPT6L, Pol II, and NRPE1.

e: Heatmap showed the enrichment of different genomic features within six defined genomic states.

f: Yesat-two hybrid assay to examine the direct interaction between SPT6L and multiple Pol V subunits. Growth of transformed yeast is shown on permissive $SD^{–His–Leu–Trp}$ plus

3AT medium. For each pair of AD and BD constructs, three different dilutions (x1, x10, and x100) were shown.

g: Immunoblot assessing the levels of Pol V subunits in the yeast. The AD represents the yeast cells containing empty AD vector, which serves as negative control. The coding sequences of all the indicated Pol V subunits were inserted into AD vector and expressed in AH109 yeast cells. Immunoblots use anti-HA (Vazyme, RA1004) antibody.

h: Confocal images examined the nuclear localization of NRPE4-GFP and NRPE7-GFP in 7 DAG roots; Scale bars, 20 μm.

Supplementary Fig. 3: Pol V is required for the intergenic enrichment of SPT6L

a: ChIP–qPCR showing genomic occupancy by NRPE1-GFP fusion protein in *NRPE1-GFP* and *spt6l NRPE1-GFP*. All the fold changes are relative to ChIP signal obtained at *ACT7* in each sample and replicates. Error bars are presented as mean values ± s.d. from three biological replicates. All significant differences were indicated with $^{*}P < 0.05$, $^{**}P < 0.01$ (unpaired, two-tailed Student's *t*-test).

b: Immunoblot assessing the levels of NRPE1 proteins in the genetic backgrounds as indicated. H3 levels served as loading controls. Data from two biological replicates were shown.

c: ChIP–qPCR showing genomic occupancy by SPT6L-GFP fusion protein in *SPT6L-GFP* and *nrpe1 SPT6L-GFP*. All the fold changes are relative to ChIP signal obtained at *ACT7* in each sample and replicates. Error bars are presented as mean values ± s.d. from three biological replicates. All significant differences were indicated with $^{*}P < 0.05$, $^{**}P < 0.01$ (unpaired, two-tailed Student's *t*-test).

d: Immunoblot assessing the levels of SPT6L proteins in the genetic backgrounds as indicated. H3 levels served as loading controls. Data from two biological replicates were shown.

Supplementary Fig. 4: SPT6L is involved in the regulation of DNA methylation

a: Volcano plot highlighted the differentially expressed genes (DGEs)between WT and *spt6l*. The increased and decreased genes in *spt6l* were labelled with red and blue, respectively. Grey dots represented the genes were failed to pass the threshold ($|\text{Fold Change}| \geq 2$ and adjust *p*-value < 0.01. Three biological replicates were included.

b: Heatmap of methylation levels of indicated mutants within *spt6l* CG, CHG, and CHH hypomethylation DMRs. Genotypes (columns) have also been clustered. The plotted values were the relative methylation level in mutants to that in WT.

Supplementary Fig. 5: Stable transgenic line of *spt6l SPT6LΔWG/GW-GFP*

a: Confocal image examined the localization of SPT6L-GFP and SPT6LΔWG/GW-GFP in 7-day after germination (DAG) roots; Scale bars, 20 μm.

b: Immunoblot assessing the levels of SPT6L and SPT6LΔWG/GW proteins. The image of Coomassie blue staining serves as loading control.

Supplementary Fig. 6: Protein amounts and the interaction between SPT6L and SPT5L

a: ChIP–qPCR showing genomic occupancy by SPT6L-GFP fusion protein in *SPT6L-GFP* and *spt5l SPT6L-GFP*. All the fold changes are relative to ChIP signal obtained at *ACT7* (*ACT7-QCF/R16*) in each sample and replicates. Error bars are presented as mean values ± s.d. from three biological replicates. All significant differences were indicated with $*P < 0.05$, $**P < 0.01$ (unpaired, two-tailed Student's *t*-test).

b: Immunoblot assessing the levels of SPT6L proteins in the *WT* and *spt5l* as indicated. H3 levels served as loading controls. Data from three biological replicates were shown.

c: Yesat-two hybrid assay to examine the direct interaction between SPT5L and SPT6L. The diagram on the left showed the different domains of SPT5L and designed truncations of SPT5L in following Yeast-two hybrid assays. Growth of transformed yeast is shown on permissive $SD^{–His–Leu–Trp}$ plus 3AT medium. For each pair of AD and BD constructs, three different dilutions (x1, x10, and x100) were shown.

d: Immunoblot assessing the levels of SPT6L proteins in the *WT*, *drm1 drm2,* and *nrpd1* as indicated. H3 levels served as loading controls. Data from three biological replicates were shown.

Supplementary Fig. 7: The frequencies of nucleotides on each position.

a: The relative nucleotide bias of each position started from the 5' end of GRO-seq reads (SRR5681049 and SRR5681053). The plotted reads were filtered by overlapping with Pol V peak.

b: The relative nucleotide bias of each position in the upstream and downstream 20-nt of nascent transcripts captured in NRPE1-GFP and *spt6l* NRPE1-GFP.

Supplementary Fig. 8: Pearson correlations of replicates in ChIP-seq and smRNA-seq data.

a: The correlation values were calculated based on reads numbers of different samples within all SPT6L or NRPE1 peaks.

b: The entire genome was divided into equal bins (100 bp in length) and the numbers of RNA-seq and 24-nt smRNA-seq reads from *WT* and *spt6l* were calculated in each bin. The correlation values were calculated based on reads numbers in each bin between *WT* and *spt6l*.

Supplementary Fig. 9: Pearson correlations of replicates in BS-seq data.

The entire genome was divided into equal bins (500 bp in length) and the methylation levels in three different contexts from *WT*, *nrpe1*, *spt6l*, and *nrpe1 spt6l* were calculated in each bin.

Supplementary Fig.1

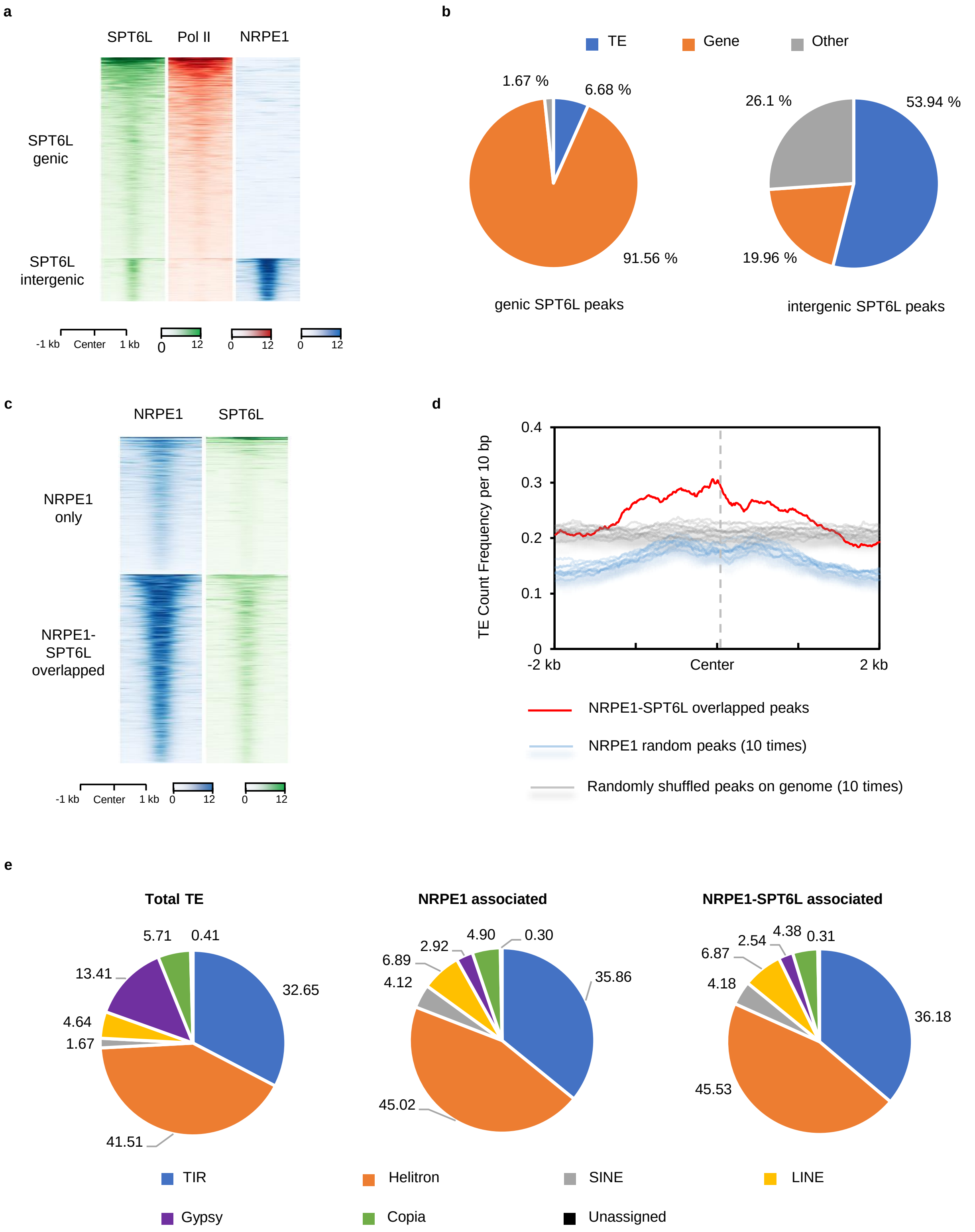

Supplementary Fig.2

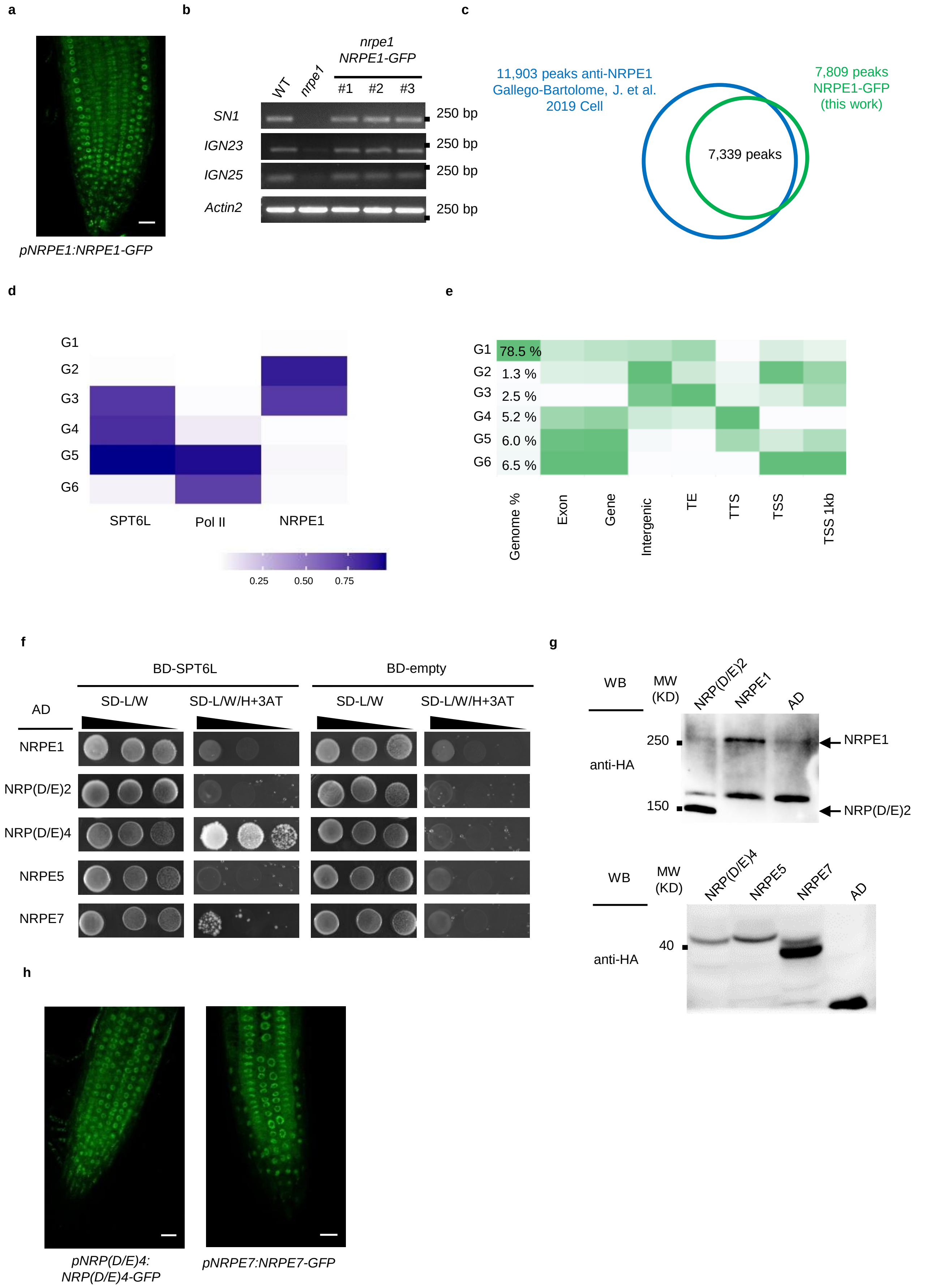

Supplementary Fig.3

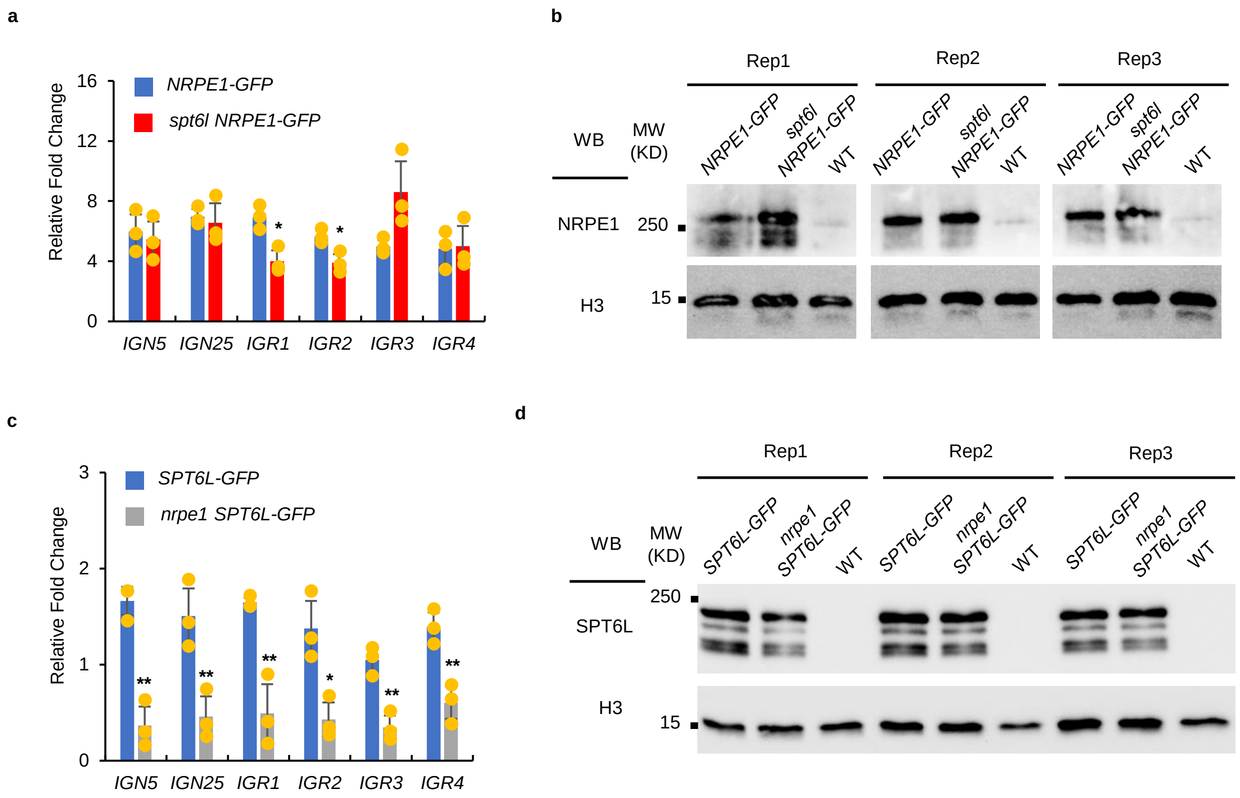

Supplementary Fig.4

a

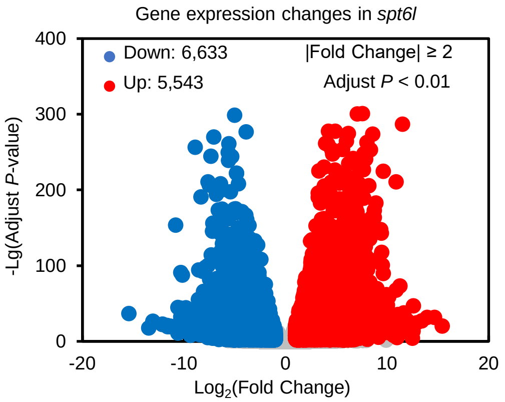

b

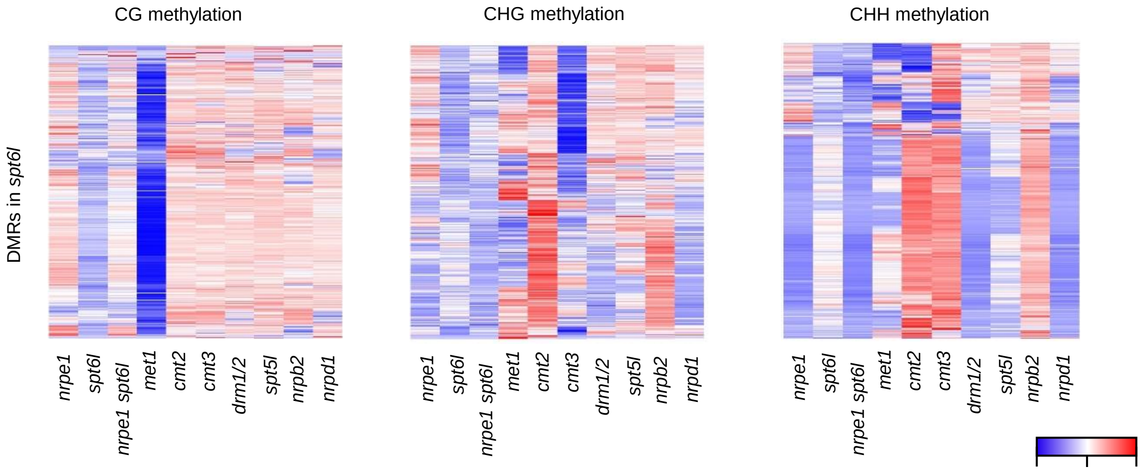

Supplementary Fig.5

a

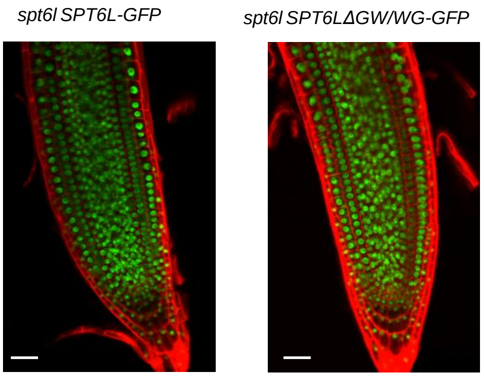

b

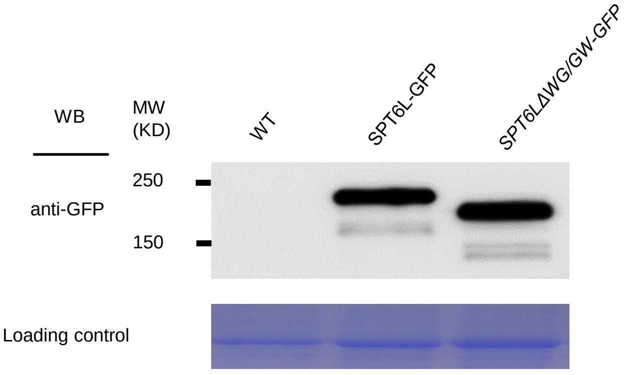

Supplementary Fig.6

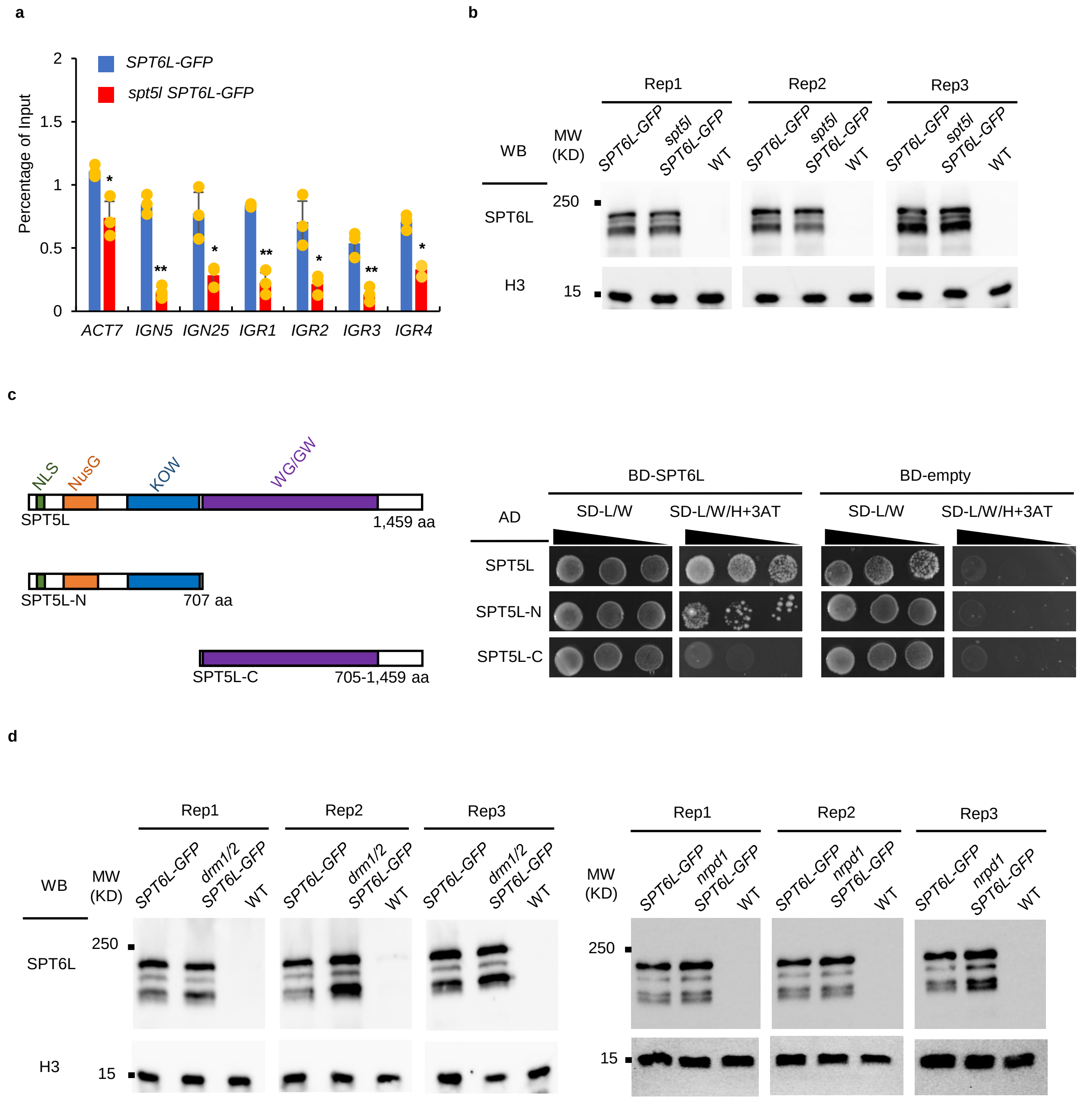

Supplementary Fig.7

a

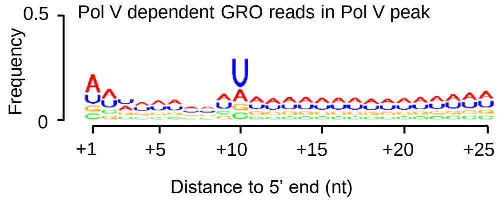

b

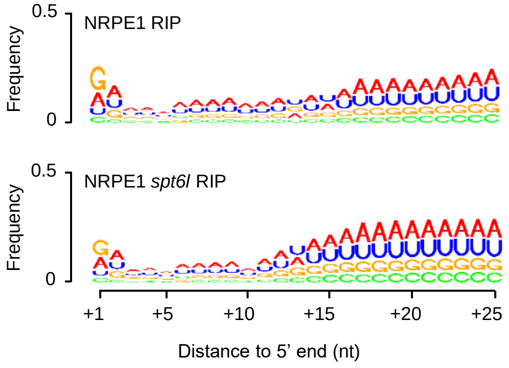

Supplementary Fig.8

a

	Rep1	Rep2
Rep1	1.00	0.90
Rep2	0.90	1.00

nrpe1 NRPE1 GFP IP

	Rep1	Rep2
Rep1	1.00	0.99
Rep2	0.99	1.00

spt6l NRPE1 GFP IP

	Rep1	Rep2
Rep1	1.00	0.73
Rep2	0.73	1.00

nrpe1 SPT6L GFP IP

	Rep1	Rep2
Rep1	1.00	0.75
Rep2	0.75	1.00

spt51 SPT6L GFP IP

	Rep1	Rep2
Rep1	1.00	0.96
Rep2	0.96	1.00

SPT6LdeltaWG/GW GFP IP

b

		WT			*spt6l*		
		Rep1	Rep2	Rep3	Rep1	Rep2	Rep3
WT	Rep1	1.00	1.00	0.99	0.56	0.53	0.49
	Rep2	1.00	1.00	0.99	0.55	0.52	0.48
	Rep3	0.99	0.99	1.00	0.54	0.51	0.47
spt6l	Rep1	0.56	0.55	0.54	1.00	0.99	0.97
	Rep2	0.53	0.52	0.51	0.99	1.00	0.98
	Rep3	0.49	0.48	0.47	0.97	0.98	1.00

RNA-seq

c

		WT			*spt6l*		
		Rep1	Rep2	Rep3	Rep1	Rep2	Rep3
WT	Rep1	1.00	0.96	0.95	0.52	0.57	0.51
	Rep2	0.96	1.00	0.98	0.53	0.58	0.51
	Rep3	0.95	0.98	1.00	0.55	0.58	0.52
spt6l	Rep1	0.52	0.53	0.55	1.00	0.98	0.99
	Rep2	0.57	0.58	0.58	0.97	1.00	0.97
	Rep3	0.51	0.51	0.52	0.99	0.97	1.00

24nt small RNA

Supplementary Fig.9

CG methylation

		WT Rep1	WT Rep2	*nrpe1* Rep1	*nrpe1* Rep2	*spt6l* Rep1	*spt6l* Rep2	*nrpe1 spt6l* Rep1	*nrpe1 spt6l* Rep2
WT	Rep1	1.00	0.99	0.97	0.97	0.97	0.98	0.97	0.97
	Rep2	0.99	1.00	0.97	0.97	0.97	0.98	0.97	0.97
nrpe1	Rep1	0.97	0.97	1.00	0.99	0.96	0.96	0.98	0.98
	Rep2	0.97	0.97	0.99	1.00	0.96	0.97	0.98	0.98
spt6l	Rep1	0.97	0.97	0.96	0.96	1.00	0.99	0.97	0.97
	Rep2	0.98	0.98	0.96	0.97	0.99	1.00	0.97	0.97
nrpe1 spt6l	Rep1	0.97	0.97	0.98	0.98	0.97	0.97	1.00	0.99
	Rep2	0.97	0.97	0.98	0.98	0.97	0.97	0.99	1.00

CHG methylation

		WT Rep1	WT Rep2	*nrpe1* Rep1	*nrpe1* Rep2	*spt6l* Rep1	*spt6l* Rep2	*nrpe1 spt6l* Rep1	*nrpe1 spt6l* Rep2
WT	Rep1	1.00	0.98	0.90	0.90	0.95	0.96	0.89	0.89
	Rep2	0.98	1.00	0.90	0.91	0.95	0.96	0.90	0.89
nrpe1	Rep1	0.90	0.90	1.00	0.98	0.91	0.91	0.97	0.97
	Rep2	0.90	0.91	0.98	1.00	0.91	0.92	0.98	0.98
spt6l	Rep1	0.95	0.95	0.91	0.91	1.00	0.97	0.92	0.92
	Rep2	0.96	0.96	0.91	0.92	0.97	1.00	0.92	0.92
nrpe1 spt6l	Rep1	0.89	0.90	0.97	0.98	0.92	0.92	1.00	0.98
	Rep2	0.89	0.89	0.97	0.98	0.92	0.92	0.98	1.00

CHH methylation

		WT Rep1	WT Rep2	*nrpe1* Rep1	*nrpe1* Rep2	*spt6l* Rep1	*spt6l* Rep2	*nrpe1 spt6l* Rep1	*nrpe1 spt6l* Rep2
WT	Rep1	1.00	0.95	0.71	0.71	0.85	0.91	0.68	0.68
	Rep2	0.95	1.00	0.70	0.71	0.86	0.93	0.68	0.68
nrpe1	Rep1	0.71	0.70	1.00	0.96	0.60	0.65	0.94	0.94
	Rep2	0.71	0.71	0.96	1.00	0.61	0.66	0.95	0.94
spt6l	Rep1	0.85	0.86	0.60	0.61	1.00	0.88	0.58	0.59
	Rep2	0.91	0.93	0.65	0.66	0.88	1.00	0.64	0.64
nrpe1 spt6l	Rep1	0.68	0.68	0.94	0.95	0.58	0.64	1.00	0.95
	Rep2	0.68	0.68	0.94	0.94	0.59	0.64	0.95	1.00

Table 1 Primers

Name	Primer Sequences (5' to 3')	Usage
NRPE1-Nde1	GGTCACGCATATGAACCTGTTGGAGTATTGGC	Yeast-two hybrid assays
NRPE1-Xho1	CCGCTCGAGTTATGTCTGCGTCTGGGACGG	Yeast-two hybrid assays
Va-Nde1-NRPE1	GTACCAGATTACGCTCATATGATGGAGGAAGAATCTACAT	Yeast-two hybrid assays
Va-NRPE1-3140	GATGCATGACGATTCCGGATCCACGTTGTCATGTCTGAACT	Yeast-two hybrid assays
Va-Nde1-SPT5L-F	GTACCAGATTACGCTCATATGGATCGCAAGGGAAAGGG	Yeast-two hybrid assays
Va-Sma1-SPT5L-R	CCGTATCGATGCCCACCCGGGTTACCATCCGGTTTTTTTGTC	Yeast-two hybrid assays
Va-SPT5L-1R	CCGTATCGATGCCCACCCGGGCCTCCTATATTCCAGTTACC	Yeast-two hybrid assays
Va-SPT5L-2F	GGTAACTGGAATATAGGAGGCCCTTCTACTGACTC	Yeast-two hybrid assays
Va-SPT5LCF	GTACCAGATTACGCTCATATGGGAGGCCCTTCTACTGACTC	Yeast-two hybrid assays
Va-SPT5L-2R	CCGTATCGATGCCCACCCGGGCTCAGAATTACCGAAGGAGC	Yeast-two hybrid assays
va-SPT5L-3F	CTTCGGTAATTCTGAGGATCCGGCTCCATGGAG	Yeast-two hybrid assays
va-NRPE7-F	GCCATGGAGGCCAGTGAATTCATGTTTCTCAAAGTCCAATTAC	Yeast-two hybrid assays
va-NRPE7-R	CAGCTCGAGCTCGATGGATCCTCACTCTTCAGATAATGG	Yeast-two hybrid assays
va-NRPE2-F	GCCATGGAGGCCAGTGAATTCATGCCAGATATGGACATTGA	Yeast-two hybrid assays
va-NRPE2-R	CAGCTCGAGCTCGATGGATCCTCAGCATAGCTTGGTGTC	Yeast-two hybrid assays
va-NRPE4-F	GCCATGGAGGCCAGTGAATTCATGTCAGAGAAAGGAGGC	Yeast-two hybrid assays
va-NRPE4-R	CAGCTCGAGCTCGATGGATCCTCATTCCGATTTCTTCAGC	Yeast-two hybrid assays
va-NRPE5-F	GCCATGGAGGCCAGTGAATTCATGGAAGTGAAAGGG	Yeast-two hybrid assays
va-NRPE5-R	CAGCTCGAGCTCGATGGATCCTCACCACACACATCGGAAG	Yeast-two hybrid assays
BD-SPT6L-F	CATGGAGGCCGAATTCATGGCGAGGAACGCAATCTC	Yeast-two hybrid assays
BD-SPT6L-R	GCAGGTCGACGGATCCTCACCATCCACCACCACCG	Yeast-two hybrid assays
NRPE1-ASC1-R	TTGGCGCGCCTTGTCTGCGTCTGGGACGGA	*pNRPE1:NRPE1-GFP* transgenic line
NRPE1-Pme1-F	TTCGTTTAAACAAACCCGGGCAGGTTGTTCATCGCA	*pNRPE1:NRPE1-GFP* transgenic line
Va107-NRPE1-R2	GCGATGAACAACCTGCCCGGGTTTAAGCTCAGTGTGCCCC	*pNRPE1:NRPE1-GFP* transgenic line
Va107-NRPE1-F2	TGTCAAACACTGATAGTTTAAACGAGTAGAGAAAGCAGATAGTC	*pNRPE1:NRPE1-GFP* transgenic line
NRPE4-Pme1-F	AGGTTCGTTTAAACAATTGAGATCCCACATCCTTTGG	*pNRPE4:NRPE4-GFP* transgenic line
NRPE4-ASC1-R	TTGGCGCGCCTTTCCGATTTCTTCAGCTTGGATAG	*pNRPE4:NRPE4-GFP* transgenic line
NRPE7-Pme1-F	AGGTTCGTTTAAACCTCTTAACCCTGTGAGAAGAAATC	*pNRPE7:NRPE7-GFP* transgenic line
NRPE7-ASC1-R	TTGGCGCGCCTCTCTTCAGATAATGGTCCAAGAT	*pNRPE7:NRPE7-GFP* transgenic line
SPT5L-Pme1-F	AGGTTCGTTTAAACCGTCAGCTCCAGTGAATCTGAATGAC	*pSPT5L:SPT5L-GFP* transgenic line
SPT5L-ASC-R1	TTGGCGCGCCTTCCTATCGCCCAGTCTACCATACGA	*pSPT5L:SPT5L-GFP* transgenic line
Va107-SPT5L-F2	ATGGTAGACTGGGCGATAGGAGCAGGGACGTCAAGTGAG	*pSPT5L:SPT5L-GFP* transgenic line
Va107-SPT5L-R2	GCCCCCCCTCGAGGCGCGCCTCCATCCGGTTTTTTTGTCAC	*pSPT5L:SPT5L-GFP* transgenic line
IGN25-ChIP-F	TGGAGCCCAAACCCACCTCTT	ChIP-qPCR
IGN25-ChIP-R	GTGTGGGCTTGGCCTCTTGGT	ChIP-qPCR
IGN5-ChIP-F	GTATCATGCGGCCCAATAACC	ChIP-qPCR
IGN5-ChIP-R	TGGGCCGAATAACAGCAAGTC	ChIP-qPCR
ACT7-F	TGTTTAGCATGCGTTGTGGTTT	ChIP-qPCR
ACT7-R	GATCGATCCAACAAGCACGG	ChIP-qPCR
IGR1-F	CGATTCACCAGAACACATAGC	ChIP-qPCR

**Name**	**Primer Sequences (5' to 3')**	**Usage**
IGR1-R	CTTGTTATCAGAAGACCTGCA	ChIP-qPCR
IGR2-F	GGCTTTGAATGTGATCGAGCCGTAC	ChIP-qPCR
IGR2-R	ACACCTGCCCCTTTCGTATCGAATGT	ChIP-qPCR
IGR3-F	GCTTACCAGCTAAGAGCATCTTC	ChIP-qPCR
IGR3-R	GAGAGAGTAGAACTCTGAGAAACCC	ChIP-qPCR
IGR4-F	GTTTCTAATAGACTGCGATGGCCAGTC	ChIP-qPCR
IGR4-R	GGGGGTTTTACGGACCAATCCAAATTG	ChIP-qPCR
ACT7-QCF16	GTATCGGGTGACAATGCAGC	ChIP-qPCR (Supplementary Figure 6A)
ACT7-QCR16	TCTTGTTTCGCATCAGAATGGT	ChIP-qPCR (Supplementary Figure 6A)
AtSN1-F	ACCAACGTGCTGTTGGCCCAGTGGTAAATC	Chop-PCR
AtSN1-R	AAAATAAGTGGTGGTTGTACAAGC	Chop-PCR
IGN5-F	TCCCGAGAAGAGTAGAACAAATGCTAAAA	Chop-PCR
IGN5-R	CTGAGGTATTCCATAGCCCCTGATCC	Chop-PCR
IGN23-F	ACTGAAAATTGTAAACAAAGAAACGGCACTACA	Chop-PCR
IGN23-R	GATCGGTCCATAAACTTGTTGGGTTT	Chop-PCR
IGN25-F	CTTCTTATCGTGTTACATTGAGAACTCTTTCC	Chop-PCR
IGN25-R	ATTCGTGTGGGCTTGGCCTCTT	Chop-PCR
Actin-F	CGAGCAGGAGATGGAAACCTCAAA	Chop-PCR
Actin-R	AAGAATGGAACCACCGATCCAGACA	Chop-PCR
FLO-P5	AATGATACGGCGACCACCGAGATCTACACTCTTTCCCTACACGACGCTCTTCCG	RIP reverse transcription and library prepration
P7	CAAGCAGAAGACGGCATACGA*G	RIP reverse transcription and library prepration
TSO-Biotin	/biotin/CCTACACGACGCTCTTCCGATCTNNNNNNNNTATA/rG//rG//rG/	RIP reverse transcription and library prepration
RTO-dT-1	CAAGCAGAAGACGGCATACGAGATTAGTGCGTGACTGGAGTTCAGACGTGTG CTCTTCCGATCTTTTTTTTTTTTTTTTTTTTTTTTTTTTTTTVN	RIP reverse transcription and library prepration
RTO-dT-2	CAAGCAGAAGACGGCATACGAGATACATCGGTGACTGGAGTTCAGACGTGTG CTCTTCCGATCTTTTTTTTTTTTTTTTTTTTTTTTTTTTTTTVN	RIP reverse transcription and library prepration
RTO-dT-3	CAAGCAGAAGACGGCATACGAGATCACTGTGTGACTGGAGTTCAGACGTGTG CTCTTCCGATCTTTTTTTTTTTTTTTTTTTTTTTTTTTTTTTVN	RIP reverse transcription and library prepration
LBb1.3	GCGTGGACCGCTTGCTGCAACT	Genotyping
nrpe1-11 LP	ATTTCTTCTTTGATGGGGGAG	Genotyping
nrpe1-11 RP	TGTCGTGGATATGACCATTTG	Genotyping
spt5l-1 LP	GTGGGAAAGGAGAAGGATCAG	Genotyping
spt5l-1 RP	AAACGCATGAAAACAAACCTG	Genotyping
spt6l LP	TGAAAAACACACAAAATCCAGG	Genotyping
spt6l RP	GATGAGCTTGAGGATGCAAAG	Genotyping
nrpd1-3 LP	TGGGTTTGCCATTTTCATATC	Genotyping
nrpd1-3 RP	GCATGCTTGAGTAAAAGGTGC	Genotyping
drm1-2 LP	CCTGTGTTGATTGGGATTCAG	Genotyping
drm1-2 RP	GTCGATGGAGTGCAACTTCTC	Genotyping
drm2-2 LP	AGATCGCTTCCAGAGTTAGCC	Genotyping
drm2-2 RP	TTGTCGCAAAAAGCAAAAGAG	Genotyping

Table 2 Sample Reads

Sample Name	Experiments	Raw Reads	Mapped Reads	Unique Reads
nrpe1 SPT6LGFP IP rep1	ChIP-seq	23,676,688	12,762,417	8,969,386
nrpe1 SPT6LGFP IP rep2	ChIP-seq	15,535,807	16,576,406	11,613,400
nrpe1 SPT6LGFP Input	ChIP-seq	25,783,122	22,214,124	16,035,492
spt5l SPT6LGFP IP rep1	ChIP-seq	14,772,194	10,698,436	6,311,658
spt5l SPT6LGFP IP rep2	ChIP-seq	16,725,091	9,772,949	7,855,647
nrpe1 NRPE1GFP IP rep1	ChIP-seq	17,908,081	11,311,143	8,198,947
nrpe1 NRPE1GFP IP rep2	ChIP-seq	11,365,343	9,172,993	7,058,827
nrpe1 NRPE1GFP Input	ChIP-seq	12,694,834	10,606,821	8,405,262
spt6l NRPE1GFP IP rep1	ChIP-seq	12,043,507	9,640,411	7,075,553
spt6l NRPE1GFP IP rep2	ChIP-seq	13,037,403	10,050,642	7,018,557
spt6l NRPE1GFP Input	ChIP-seq	16,061,083	13,895,917	10,966,087
SPT6LdeltaWG/GWGFP IP rep1	ChIP-seq	6,955,345	3,998,674	2,698,982
SPT6LdeltaWG/GWGFP IP rep2	ChIP-seq	10,089,591	6,230,992	3,681,226
SPT6LdeltaWG/GWGFP Input	ChIP-seq	9,576,070	9,097,324	5,899,925
WT rep1	RNA-seq	52,799,230	49,590,526	/
WT rep2	RNA-seq	49,369,979	46,630,456	/
WT rep3	RNA-seq	49,682,669	46,724,181	/
spt6l rep1	RNA-seq	44,153,861	40,973,341	/
spt6l rep2	RNA-seq	48,410,372	45,216,982	/
spt6l rep3	RNA-seq	41,145,507	38,547,238	/
WT rep1	smRNA-seq	11,895,434	4,464,608	/
WT rep2	smRNA-seq	11,669,508	5,183,120	/
WT rep3	smRNA-seq	13,939,858	4,905,388	/
spt6l rep1	smRNA-seq	11,461,290	5,062,444	/
spt6l rep2	smRNA-seq	13,423,906	5,550,727	/
spt6l rep3	smRNA-seq	11,318,669	3,983,365	/
WT rep1	BS-seq	21,802,396	/	11,280,767
WT rep2	BS-seq	33,769,639	/	19,077,922
spt6l rep1	BS-seq	17,125,158	/	9,083,920
spt6l rep2	BS-seq	33,751,600	/	18,503,804
nrpe1 rep1	BS-seq	21,768,070	/	10,916,201
nrpe1 rep2	BS-seq	34,502,359	/	17,838,894
nrpe1 spt6l rep1	BS-seq	32,901,073	/	18,852,875
nrpe1 spt6l rep2	BS-seq	31,583,622	/	16,958,685
spt6l SPT6LdeltaWG/GW	BS-seq	20,695,174	/	10,778,207
nrpe1 RIP	RIP-seq	4,984,248	/	4,037,076
nrpe1 NRPE1GFP RIP	RIP-seq	10,535,572	/	8,415,015
spt6l nrpe1 NRPE1GFP RIP	RIP-seq	10,744,978	/	8,770,763
nrpe1 RIP rep2	RIP-seq	1,571,955	/	357,858
nrpe1 NRPE1GFP RIP rep2	RIP-seq	2,170,018	/	1,106,398

Sample Name	Experiments	Raw Reads	Mapped Reads	Unique Reads
spt6l nrpe1 NRPE1GFP RIP rep2	RIP-seq	8,588,635	/	4,023,827
