# Supplementary material for: A conserved Pol II elongator SPT6L mediates Pol V transcription to regulate RNA-directed DNA methylation in Arabidopsis
Source: Nat Commun. 2024 May 25;15:4460. doi: 10.1038/s41467-024-48940-8 (PMC11127964; doi:10.1038/s41467-024-48940-8)
Supplement: Supplementary file 3 — Description of additional supplementary files [file 41467_2024_48940_MOESM3_ESM.pdf]

Description of Additional Supplementary Files

Supplementary Data 1 - NRPE1 and NRPE1-SPT6L overlapped peaks

Supplementary Data 2 – The six genomic states defined by HMM

Supplementary Data 3 – Genomic states overlapped genes

Supplementary Data 4 – RNA-seq data with expression changed genes and TEs

Supplementary Data 5 - Pol V peaks clustered by slicing preference of U-10
