# Supplementary material for: A conserved Pol II elongator SPT6L mediates Pol V transcription to regulate RNA-directed DNA methylation in Arabidopsis
Source: Nat Commun. 2024 May 25;15:4460. doi: 10.1038/s41467-024-48940-8 (PMC11127964; doi:10.1038/s41467-024-48940-8)
Supplement: Supplementary file 9 — Reporting Summary [file 41467_2024_48940_MOESM9_ESM.pdf]

nature portfolio

Corresponding author(s): Chen Chen

Last updated by author(s): Apr 22, 2024

Reporting Summary

Nature Portfolio wishes to improve the reproducibility of the work that we publish. This form provides structure for consistency and transparency in reporting. For further information on Nature Portfolio policies, see our Editorial Policies and the Editorial Policy Checklist.

Statistics

For all statistical analyses, confirm that the following items are present in the figure legend, table legend, main text, or Methods section.

n/a	Confirmed	
☐	☒	The exact sample size (*n*) for each experimental group/condition, given as a discrete number and unit of measurement
☐	☒	A statement on whether measurements were taken from distinct samples or whether the same sample was measured repeatedly
☐	☒	The statistical test(s) used AND whether they are one- or two-sided<br>*Only common tests should be described solely by name; describe more complex techniques in the Methods section.*
☒	☐	A description of all covariates tested
☒	☐	A description of any assumptions or corrections, such as tests of normality and adjustment for multiple comparisons
☐	☒	A full description of the statistical parameters including central tendency (e.g. means) or other basic estimates (e.g. regression coefficient) AND variation (e.g. standard deviation) or associated estimates of uncertainty (e.g. confidence intervals)
☐	☒	For null hypothesis testing, the test statistic (e.g. *F*, *t*, *r*) with confidence intervals, effect sizes, degrees of freedom and *P* value noted<br>*Give P values as exact values whenever suitable.*
☒	☐	For Bayesian analysis, information on the choice of priors and Markov chain Monte Carlo settings
☒	☐	For hierarchical and complex designs, identification of the appropriate level for tests and full reporting of outcomes
☒	☐	Estimates of effect sizes (e.g. Cohen's *d*, Pearson's *r*), indicating how they were calculated

Our web collection on statistics for biologists contains articles on many of the points above.

Software and code

Policy information about availability of computer code

Data collection	all the high-throughput sequencing data were generated from illumnia Novaseq platform. qPCR from LightCycler480 system.
Data analysis	All the softwares and version used in this study can be found in Bioinformatic analysis section. There is no in-house code in this study.

For manuscripts utilizing custom algorithms or software that are central to the research but not yet described in published literature, software must be made available to editors and reviewers. We strongly encourage code deposition in a community repository (e.g. GitHub). See the Nature Portfolio guidelines for submitting code & software for further information.

Data

Policy information about availability of data

All manuscripts must include a data availability statement. This statement should provide the following information, where applicable:

- Accession codes, unique identifiers, or web links for publicly available datasets
- A description of any restrictions on data availability
- For clinical datasets or third party data, please ensure that the statement adheres to our policy

All the raw data and processed data can be reached in the Gene Expression Omnibus (GEO) under accession GSE233781. Plotted regions can be found in supplementary datasets 1-5

Research involving human participants, their data, or biological material

Policy information about studies with human participants or human data. See also policy information about sex, gender (identity/presentation), and sexual orientation and race, ethnicity and racism.

Reporting on sex and gender	*Use the terms sex (biological attribute) and gender (shaped by social and cultural circumstances) carefully in order to avoid confusing both terms. Indicate if findings apply to only one sex or gender; describe whether sex and gender were considered in study design; whether sex and/or gender was determined based on self-reporting or assigned and methods used. Provide in the source data disaggregated sex and gender data, where this information has been collected, and if consent has been obtained for sharing of individual-level data; provide overall numbers in this Reporting Summary. Please state if this information has not been collected. Report sex- and gender-based analyses where performed, justify reasons for lack of sex- and gender-based analysis.*
Reporting on race, ethnicity, or other socially relevant groupings	*Please specify the socially constructed or socially relevant categorization variable(s) used in your manuscript and explain why they were used. Please note that such variables should not be used as proxies for other socially constructed/relevant variables (for example, race or ethnicity should not be used as a proxy for socioeconomic status). Provide clear definitions of the relevant terms used, how they were provided (by the participants/respondents, the researchers, or third parties), and the method(s) used to classify people into the different categories (e.g. self-report, census or administrative data, social media data, etc.) Please provide details about how you controlled for confounding variables in your analyses.*
Population characteristics	*Describe the covariate-relevant population characteristics of the human research participants (e.g. age, genotypic information, past and current diagnosis and treatment categories). If you filled out the behavioural & social sciences study design questions and have nothing to add here, write "See above."*
Recruitment	*Describe how participants were recruited. Outline any potential self-selection bias or other biases that may be present and how these are likely to impact results.*
Ethics oversight	*Identify the organization(s) that approved the study protocol.*

Note that full information on the approval of the study protocol must also be provided in the manuscript.

Field-specific reporting

Please select the one below that is the best fit for your research. If you are not sure, read the appropriate sections before making your selection.

☒ Life sciences ☐ Behavioural & social sciences ☐ Ecological, evolutionary & environmental sciences

For a reference copy of the document with all sections, see nature.com/documents/nr-reporting-summary-flat.pdf

Life sciences study design

All studies must disclose on these points even when the disclosure is negative.

Sample size	For all the ChIP-seq, BS-seq, RIP-seq, RNA-seq and siRNA-seq, at least 100 10-day-old seedlings were collected for each sample. The qPCR, immunoblotting, and Co-IP used at least 50 seedlings for each replicate.
Data exclusions	No data exclusion
Replication	Three biological replicates were used for qPCR, ChIP-qPCR, smRNA-seq, RNA-seq and immunoblotting. Two biological replicates were used for ChIP-seq, BS-seq, Co-IP, and RIP-seq. all attempts at replication were successful.
Randomization	wild-type and mutant plants were grown side by side on the same growth conditions. The plants were then randomly collected for experiments.
Blinding	Experiments were not blinded. Data were always collected according to the genotype of plants.

Reporting for specific materials, systems and methods

We require information from authors about some types of materials, experimental systems and methods used in many studies. Here, indicate whether each material, system or method listed is relevant to your study. If you are not sure if a list item applies to your research, read the appropriate section before selecting a response.

Materials & experimental systems

n/a	Involved in the study
☐	☒ Antibodies
☒	☐ Eukaryotic cell lines
☒	☐ Palaeontology and archaeology
☒	☐ Animals and other organisms
☒	☐ Clinical data
☒	☐ Dual use research of concern
☐	☒ Plants

Methods

n/a	Involved in the study
☐	☒ ChIP-seq
☒	☐ Flow cytometry
☒	☐ MRI-based neuroimaging

Antibodies

Antibodies used	anti-GFP (Abcam, ab290); anti-FLAG M2 Magnetic Beads (Sigma-Aldrich, M8823); anti-GFP nanobody agarose beads (KTHEALTH, KTSM1301); anti-H3 (Abcam, ab1791); anti-MYC (Abcam, ab9106); anti-HA (Vazyme, RA1004; 1:5,000 dilution); anti-GFP (Yeasen, 31002ES60; 1:10,000 dilution)
Validation	anti-GFP (Abcam, ab290); https://www.abcam.com/products/primary-antibodies/gfp-antibody-ab290.html<br>anti-FLAG M2 Magnetic Beads (Sigma-Aldrich, M8823); https://www.sigmaaldrich.cn/CN/zh/product/sigma/m8823<br>anti-GFP nanobody agarose beads (KTHEALTH, KTSM1301); http://www.ktsm-life.com/product-detail-260.html<br>anti-H3 (Abcam, ab1791); https://www.abcam.com/products/primary-antibodies/histone-h3-antibody-nuclear-marker-and-chip-grade-ab1791.html<br>anti-MYC (Abcam, ab9106);https://www.abcam.com/products/primary-antibodies/myc-tag-antibody-ab9106.html

Dual use research of concern

Policy information about dual use research of concern

Hazards

Could the accidental, deliberate or reckless misuse of agents or technologies generated in the work, or the application of information presented in the manuscript, pose a threat to:

No	Yes
☒	☐ Public health
☒	☐ National security
☒	☐ Crops and/or livestock
☒	☐ Ecosystems
☒	☐ Any other significant area

Experiments of concern

Does the work involve any of these experiments of concern:

No	Yes
☒	☐ Demonstrate how to render a vaccine ineffective
☒	☐ Confer resistance to therapeutically useful antibiotics or antiviral agents
☒	☐ Enhance the virulence of a pathogen or render a nonpathogen virulent
☒	☐ Increase transmissibility of a pathogen
☒	☐ Alter the host range of a pathogen
☒	☐ Enable evasion of diagnostic/detection modalities
☒	☐ Enable the weaponization of a biological agent or toxin
☒	☐ Any other potentially harmful combination of experiments and agents

Plants

Seed stocks	The source of plant seeds were mentioned in Material and Methods sections.
Novel plant genotypes	all the transgenic plants were generated as described in Material and Methods sections.

Authentication	all the transgenic lines were validated from functional and genetic ways, which have been mentioned in Results section.

ChIP-seq

Data deposition

☒ Confirm that both raw and final processed data have been deposited in a public database such as GEO.

☒ Confirm that you have deposited or provided access to graph files (e.g. BED files) for the called peaks.

Data access links
May remain private before publication.

www.ncbi.nlm.nih.gov/geo/query/acc.cgi?acc=GSE233781

Files in database submission

GSM7434745	BS-seq nrpe1 rep1
GSM7434746	BS-seq nrpe1 rep2
GSM7434747	BS-seq nrpe1 spt6l rep1
GSM7434748	BS-seq nrpe1 spt6l rep2
GSM7434749	BS-seq spt6ldeltaWG/GW
GSM7434750	BS-seq spt6l rep1
GSM7434751	BS-seq spt6l rep2
GSM7434752	BS-seq WT rep1
GSM7434753	BS-seq WT rep2
GSM7434754	NRPE1-GFP input
GSM7434755	nrpe1 input
GSM7434756	NRPE1-GFP IP rep1
GSM7434757	NRPE1-GFP IP rep2
GSM7434758	spt6l input
GSM7434759	NRPE1-GFP spt6l IP rep1
GSM7434760	NRPE1-GFP spt6l IP rep2
GSM7434761	SPT6L-GFP nrpe1 IP rep1
GSM7434762	SPT6L-GFP nrpe1 IP rep2
GSM7434763	SPT6L-GFP spt5l IP rep1
GSM7434764	SPT6L-GFP spt5l IP rep2
GSM7434765	RIP-seq NRPE1-GFP rep1
GSM7434766	RIP-seq NRPE1-GFP spt6l rep1
GSM7434767	RIP-seq nrpe1 rep1
GSM8011919	RIP-seq NRPE1-GFP rep2
GSM8011920	RIP-seq nrpe1 rep2
GSM8011921	RIP-seq NRPE1-GFP spt6l rep2
GSM7434768	SPT6LdeltaWG/GW IP rep1
GSM7434769	SPT6LdeltaWG/GW IP rep2
GSM7434770	SPT6LdeltaWG Input
GSM7434771	smRNA-seq spt6l rep1
GSM7434772	smRNA-seq spt6l rep2
GSM7434773	smRNA-seq spt6l rep3
GSM7434774	smRNA-seq WT rep1
GSM7434775	smRNA-seq WT rep2
GSM7434776	smRNA-seq WT rep3
GSM8011913	RNA_spt6l_rep1
GSM8011914	RNA_spt6l_rep2
GSM8011915	RNA_spt6l_rep3
GSM8011916	RNA_WT_rep1
GSM8011917	RNA_WT_rep2
GSM8011918	RNA_WT_rep3

Genome browser session
(e.g. UCSC)

https://bioviz.org

Methodology

Replicates	Two biological replicates for each sample.
Sequencing depth	sequencing reads and method can be found in Supplementary Figure 7 and Method section
Antibodies	anti-GFP(ab290, Abcam)
Peak calling parameters	MACS2(2.2.7.1 -f BEDPE, -g 135000000, -q 0.001), IDR(3.5.1, --idr-threshold 0.01)
Data quality	IDR(3.5.1, --idr-threshold 0.01) to generate highly reproducible peaks. See Method section.

Software	all the used softwares are listed in the bioinformatic analysis section.
